# Medical oncologists’ perspectives of the Veterans Affairs National Precision Oncology Program

**DOI:** 10.1371/journal.pone.0235861

**Published:** 2020-07-24

**Authors:** Vishal Vashistha, Pradeep J. Poonnen, Jane L. Snowdon, Halcyon G. Skinner, Victoria McCaffrey, Neil L. Spector, Bradley Hintze, Jill E. Duffy, Dilhan Weeraratne, Gretchen P. Jackson, Michael J. Kelley, Vimla L. Patel

**Affiliations:** 1 Department of Veterans Affairs, National Precision Oncology Program, Durham, NC, United States of America; 2 Duke Cancer Institute, Durham, NC, United states of America; 3 Department of Hematology and Oncology, Durham Veterans Affairs Medical Center, Durham, NC, United States of America; 4 Watson Health, IBM, Cambridge, MA, United States of America; 5 College of Health, Lehigh University, Bethlehem, PA, United States of America; 6 Section of Surgical Sciences, Vanderbilt University Medical Center, Nashville, TN, United States of America; 7 Center for Cognitive Sciences in Medicine and Public Health, The New York Academy of Medicine, New York City, NY, United States of America; Cancer Institute, ITALY

## Abstract

**Background:**

To support the rising need for testing and to standardize tumor DNA sequencing practices within the U.S. Department of Veterans Affairs (VA)’s Veterans Health Administration (VHA), the National Precision Oncology Program (NPOP) was launched in 2016. We sought to assess oncologists’ practices, concerns, and perceptions regarding Next-Generation Sequencing (NGS) and the NPOP.

**Materials and methods:**

Using a purposive total sampling approach, oncologists who had previously ordered NGS for at least one tumor sample through the NPOP were invited to participate in semi-structured interviews. Questions assessed the following: expectations for the NPOP, procedural requirements, applicability of testing results, and the summative utility of the NPOP. Interviews were assessed using an open coding approach. Thematic analysis was conducted to evaluate the completed codebook. Themes were defined deductively by reviewing the direct responses to interview questions as well as inductively by identifying emerging patterns of data.

**Results:**

Of the 105 medical oncologists who were invited to participate, 20 (19%) were interviewed from 19 different VA medical centers in 14 states. Five recurrent themes were observed: (1) Educational Efforts Regarding Tumor DNA Sequencing Should be Undertaken, (2) Pathology Departments Share a Critical Role in Facilitating Test Completion, (3) Tumor DNA Sequencing via NGS Serves as the Most Comprehensive Testing Modality within Precision Oncology, (4) The Availability of the NPOP Has Expanded Options for Select Patients, and (5) The Completion of Tumor DNA Sequencing through the NPOP Could Help Improve Research Efforts within VHA Oncology Practices.

**Conclusion:**

Medical oncologists believe that the availability of tumor DNA sequencing through the NPOP could potentially lead to an improvement in outcomes for veterans with metastatic solid tumors. Efforts should be directed toward improving oncologists’ understanding of sequencing, strengthening collaborative relationships between oncologists and pathologists, and assessing the role of comprehensive NGS panels within the battery of precision tests.

## Introduction

Approaches to systemic cancer treatment are evolving with the widespread growth of precision oncology. Particularly among patients with metastatic disease, the use of targeted therapies and immunotherapy have improved outcomes for a growing number of cancers while mitigating the toxicities often associated with traditional cytotoxic chemotherapy [1]. The likelihood of response to these novel treatments is frequently associated with biomarkers identified by DNA sequencing of tumor samples. As such, great efforts to prepare samples, conduct tumor DNA sequencing, and deliver the findings and interpretations to oncologists have been undertaken within the molecular pathology departments of major cancer centers and by external vendor molecular laboratories [2].

Of the varying forms of precision testing, next-generation sequencing (NGS) of multi-gene panels is increasingly becoming the standard modality for tumor DNA sequencing. NGS panels provide opportunities to assess the mutational status of a large number of genes and carry the potential to routinely conduct sequencing of a patient’s whole exome or entire genome, simultaneously. The number of molecular targets and biomarkers that may be determined by a multi-gene NGS panel is exponentially greater than with other forms of precision testing, namely quantitative polymerase chain reaction (QPCR), which facilitates single mutation detection, and immunohistochemical staining (IHC), but the cost and required time to complete an expansive NGS panel is far greater. The resources and personnel to complete NGS of tumor samples is also often unavailable at many hospitals.

Though multi-gene NGS panels output a large amount of molecular information, which is considered comprehensive within the growing arena of precision oncology, the vast majority of identified mutations are not amenable to available treatments at this time. In fact, the utility of conducting multi-gene NGS panels across all advanced tumor types has been questioned previously. The concerns are secondary to the costs of tumor DNA sequencing despite limited evidence supporting its use. A few studies have evaluated the benefits of conducting multi-gene NGS panels in a widespread fashion [3–6]. A limited benefit has been identified in altering treatment patterns and identifying germline mutations. To date, an evaluation of improvements in survival has not been conducted. Nevertheless, the broad implementation of this testing modality across tumor types remains controversial.

For these reasons, The National Comprehensive Cancer Network (NCCN) provides recommendations for conducting tumor DNA sequencing via multi-gene NGS panels on a case-by-case basis [7]. The disease characteristics, previous lines of therapy, and performance status and preferences of each patient should be taken into account prior to completing testing. Nevertheless, the indications for ordering tumor DNA sequencing will continue to grow as the number of investigational and approved targeted therapies expands [8]. Consequently, medical oncologists are increasingly tasked with identifying patients who would benefit from tumor DNA sequencing, detailing the utility of sequencing with their patients, coordinating the submission of samples for NGS, and prescribing therapies based upon the results. Tumor DNA sequencing is a relatively new clinical tool for many oncologists, and the growing integration of this tumor-agnostic test into practice likely leads to a considerable number of difficulties related to time and implementation.

To facilitate the rising demand for testing and to standardize tumor DNA sequencing practices within the U.S. Department of Veterans Affairs (VA)’s Veterans Health Administration (VHA), the National Precision Oncology Program (NPOP) was launched in 2016. The NPOP provides opportunities for veterans throughout the country to have access to targeted cancer care by supporting the completion of NGS through an external vendor molecular laboratory and by facilitating consultative guidance from experts in cancer genomics based upon the results of testing. Oncologists serving within VHA practices from diverse settings may submit tumor samples for sequencing and prescribe targeted therapies thereafter with the aid of the vendor’s automated interpretive summaries and the program’s national consult service. Over 6000 samples from veterans have undergone NGS [9]. VHA oncologists have developed a growing understanding of the testing process, reported results, and limitations of tumor DNA sequencing through the program.

Previous qualitative studies have assessed the perceptions of tumor DNA sequencing among patients and providers. A substantial number of unintended real-world consequences have been discovered with the accumulating use of comprehensive NGS panels. Patients are increasingly asked to handle complex genetic findings, which may be unrelated to their primary malignancies but convey greater risk for other tumors or non-malignant conditions [10–12]. Genetic counselors need more familiarity with molecular biology to discuss unforeseen mutations with referred patients [13, 14]. Lastly, pathologists and genomic experts are facing difficulties toward standardizing testing practices at facilities across the world [15]. To the best of our knowledge, a semi-structured interview-based study among medical oncologists regarding the expanding use of NGS to identify clinically relevant somatic mutations across tumor types has not been conducted. Such an evaluation is warranted as medical oncologists are chiefly responsible for requesting testing and taking actions based upon the results.

As such, we designed and deployed semi-structured interviews to assess the concerns, practices, and perceptions regarding tumor DNA sequencing and the NPOP among VHA oncologists who have participated in the program. Though the oncologists included in our study were uniquely employed by the VHA, the implications of our findings are relatable to providers in other settings. The indications for tumor DNA sequencing and the associated external vendor molecular laboratories are similar for oncologists serving within University-based medical centers and private cancer practices.

## Materials and methods

### Design

A semi-structured interview protocol was created following discussions with expert oncologists and included the authors’ previous experiences with ordering tumor DNA sequencing and overseeing the NPOP. Questions were constructed to assess the following domains: expectations for the NPOP, procedural requirements to complete tumor DNA sequencing, applicability of testing results, and the summative utility of the NPOP. An experienced specialist (VP) within the field of qualitative investigations guided the development and flow of questions. Prior to conducting recorded interviews with recruited participants, two pilot interviews were completed to assess the quality and delivery of questions.

### Recruited participants

The inclusion criteria for invited participants specified that each interviewee (1) was a specialist in the field of medical oncology, (2) practiced within the VHA at the time of interview, and (3) had submitted at least one sample for tumor DNA sequencing through the NPOP. The exclusion criteria restricted other providers within the field of oncology from participating. Purposive sampling was undertaken with a total population sampling approach. Invitations were disseminated via e-mail and by communication with VHA oncologists at the annual meeting for The Association of VA Hematology and Oncology (AVAHO) in 2018. In total, 105 medical oncologists were requested to participate from all geographical areas of the country and from practices of varying sizes.

### Data collection

The background of the interviewer was an experienced fellow in the fields of hematology and oncology. Semi-structured interviews, both in-person and telephonic, frequently lasting between 15 and 30 minutes with a mean duration of 17 minutes and 10 seconds, were scheduled and completed with interested oncologists. Participants were interviewed individually between September 2018 and January 2019. Informed consent was obtained verbally from all included participants prior to conducting interviews. Interviewees were often asked to expound on their opinions and attitudes from their structured responses by specifically describing their previous experiences with tumor DNA sequencing. Audio of all interviews was recorded and professionally transcribed by a transcriptionist who was not associated with the study team. Prior to conducting interviews with recruited participants, two pilot interviews were completed to assess the quality and delivery of questions.

### Data analysis

Data organization, management, interview question response tracking, and coding was performed using Microsoft Excel. Five representative verbatim transcripts were initially reviewed and responses were assessed by two study team members through open coding. All team members reviewed the five coded transcripts to identify differences among codes between the two sets of transcripts and to reach a consensus for each difference. The remaining interviews were coded thereafter by one study team member under the consideration of previous recommendations from all team members for nuanced excerpts of transcripts. Following review of all transcripts, thematic analysis was undertaken as previously described byBraun and Clarke et al. [16]. Patterns of data elicited by our semi-structured interview questions were evaluated while emerging themes were defined. As such, themes could be derived from the interviews by both inductive and deductive approaches. Indicative quotes representing each theme were selected for reporting.

### Regulatory

This qualitative analysis was conducted within the realm of clinical operations’ quality improvement of the VHA. Permission to publish the study’s findings was obtained from a VHA employee with supervisory authority to approve completed investigations.

## Results

Twenty interviews were conducted, by a single interviewer (VV), with oncologists treating patients at VHA practices geographically distributed across the U.S. The response rate was 19% (20/105), which is not unusual for busy oncologists. Table 1 shows the characteristics of the participants.

**Table 1:** Characteristics of interviewees.

**Gender**	N (%)
Male:	11 (55)
Female:	9 (45)
**Years in Oncology Practice**	
Completing Fellowship:	2 (10)
1 to 9:	6 (30)
10 to 19:	5 (25)
20 or more:	7 (35)
**Geographical Location of Practice in US**	
West:	5 (25)
Midwest:	4 (20)
Northeast:	6 (30)
South:	5 (25)
Practice Affiliated with Academic Medical Center*	
Yes:	17 (85)
No:	3 (15)
**Reported Number of Samples sent for NGS Annually:**	
1 to 5:	5 (25)
6 to 20:	4 (20)
21 to 49:	7 (35)
50 or more:	4 (20)

*Academic medical center affiliation indicates that medical oncologists have a dual appointment at both a VA medical center and a local academic medical center; their practice may or may not include treating patients at the affiliated academic medical center in addition to treating patients at their local VA medical center.

Following review of five transcripts by two study team members (VV and PP), the remaining study team members compared the designated codes between each author, and a consensus for the differences was reached through discussion. The remaining 15 interviews were then conducted by a single study team member (VV). With the use of open coding and a deductive approach to thematic analysis, the following four recurrent themes were identified: (1) Educational Efforts Regarding Tumor DNA Sequencing Should be Undertaken, (2) Pathology Departments Share a Critical Role in Facilitating Test Completion, (3) Tumor DNA Sequencing via NGS Serves as the Most Comprehensive Testing Modality within Precision Oncology, and (4) The Availability of the NPOP Has Expanded Options for Select Patients. A final theme, (5) The Completion of Tumor DNA Sequencing through the NPOP Could Help Improve Research Efforts within VHA Oncology Practices, was identified inductively. Quotations indicative of each theme are conveyed in Table 2.

**Table 2:** Indicative quotations from interviewed oncologists.

Theme 1: Educational Efforts Regarding Tumor DNA Sequencing Should be Undertaken	
Quote A: Interviewee 5; male, in practice for more than 20 years, estimates sending 1 sample or less for testing monthly from a VA site in midwest region of U.S.	*“Another one [of my patients]…had a number of [tumor] samples [taken]*, *and I do not know if we've been able to have sufficient tissue*. *Again*, *I don't know how much tissue is needed [to conduct sequencing]*.*”*
Quote B: Interviewee 10; female, in practice for less than 10 years, estimates sending ~2 samples for testing monthly from a VA site in midwest region of U.S.	*“Sometimes you need information sooner than it [takes to be] available…Specimen viability I think is and was always a concern*, *how old of a sample*? *is it still relevant*? *those kinds of things*.*”*
Quote C: Interviewee 3; male, in practice for less than 10 years, estimates sending between 5 and 10 samples for testing monthly from a VA site in west region of U.S.	*“[Occasionally*,*] I don't really know [what a specific] mutation is and what [is the functional consequence of that mutation…so there's a little bit more uncertainty that I'm making the right clinical decisions with the information*.* *.* *.*I felt like the information being given to me may be beyond my scope*.*”*
Quote D: Interviewee 11; female, in practice for less than 10 years, estimates sending ~2 samples for testing monthly from a VA site in midwest region of U.S.	*“[One issue is the] interpretation of some of the results*. *I think nobody knows half of all these other mutations*, *like these alternate [Androgen Receptor] mutations*, *and what do you do with that information*? *So [we are] handling information we don't really have an answer for*.*”*
Quote E: Interviewee 8; male, in final year of fellowship training, estimates sending 1 sample or less for testing monthly from a VA site in south region of U.S.	*“[Some form of technical support would be] helpful toward interpreting difficult [mutations]*, *so if there is a question on a result*, *helping to understand what that means*.*”*
Quote F: Interviewee 18; male, in practice for more than 20 years, estimates sending 1 sample or less for testing monthly from a VA site in east region of U.S.	*“I feel that there is so much expertise in those Ph*.*D*.*’s in the labs*, *[that they could answer] the questions that we are asking and struggling with…they could be drawn into [a Tumor Board]…they would [also] learn a lot*, *and that could be an inspiration…for their research*.*”*
Theme 2: Pathology Departments Share a Critical Role in Facilitating Test Completion	
Quote G: Interviewee 7; female, in practice for less than 10 years, estimates sending 1 sample or less for testing monthly from a VA site in south region of U.S.	*“Yes*, *yes*, *[Our pathologists are} very responsive…I guess [one of them] had done some consulting for a different company [involved with sequencing in the past] and [has previously provided his thoughts about optimal tumor samples for sequencing]*. *[Our pathologists] are very receptive*, *very helpful*.*”*
Quote H: Interviewee 12, male, in practice for less than 10 years, estimates sending ~2 samples for testing monthly from a VA site in east region of U.S.	*“We’re lucky…I don't even order [sequencing]*. *I just call [our pathologist] and he orders it for me so I really don't [have to be involved in sample submission]*. *[Our Chief of Pathology]…doesn't mind [submitting samples]*. *He's kind of taken upon himself to do all the ordering [following our requests]*.*”*
Quote I: Interviewee 5; male, in practice for more than 20 years, estimates sending 1 sample or less for testing monthly from a VA site in midwest region of U.S.	*“We…sat down at the very beginning with our pathologists and our lab and kind of decided a workflow for how we're going to [complete testing]…I basically make a direct request to the pathologist on the case and then they send it out*.*”*
Quote J: Interviewee 16; female, in practice for more than 20 years; estimates sending 3 to 5 samples for testing monthly from a VA site in west region of U.S.	*[Sequencing of tumor samples] has gone fairly smoothly from the solid tumor specimen point of view*. *The liquid biopsies are a little dicey*, *just because [the plasma samples] go to a different pathologist and that process is not so firmly in place*. *But part of that has to do with our phlebotomy lab being swamped and other types of things*.
Quote K: Interviewee 7; female, in practice less than 10 years, estimates sending 1 sample or less for testing monthly from a VA site in south region of U.S.	*“We've basically asked [a technician] who's in [our] molecular department to take on [preparing and submitting samples for sequencing] as additional work responsibility*. *So somebody is having workload shifted to help with this”*.
Quote L: Interviewee 5; male, in practice for more than 20 years, estimates sending 1 sample or less for testing monthly from a VA site in midwest region of U.S.	*“We have workload concerns*. *We have to be selective in whose samples we send out*. *They only have so much staff in the Pathology Department that can prepare [tumor specimens]*. *If we wanted to send [samples for each patient who qualifies for testing]…we'd have to bring somebody else*.*”*
Theme 3: Tumor DNA Sequencing via NGS Serves as the Most Comprehensive Testing Modality within Precision Oncology	
Quote M: Interviewee 6; female, in practice between 10 and 20 years, estimates sending ~1 sample for testing monthly from a VA site in east region of U.S.	*“Initially*, *I saw [completing sequencing as] a more efficient one-stop shopping way to get the basic mutations [that] we need*, *especially in lung cancer*. *The more I use it*, *the more I learn about it*, *I realize that*, *also*, *we pick up the rare mutations that are becoming more and more important*.*”*
Quote N: Interviewee 19; female, in practice between 10 and 20 years, estimates sending between 5 and 10 samples for testing monthly from a VA site in south region of U.S.	*“If we send [a sample for NGS]*, *we are [no longer] ordering EGFR [individually] locally…the results [are] very helpful*, *because we [not only detect] targetable mutations that we are looking at*, *but we can also know about the other mutations that are present*. *And they are helpful in deciding about second and third line treatment of those patients*.*”*
Quote O: Interviewee 7; female, in practice less than 10 years, estimates sending 1 sample or less for testing monthly from a VA site in south region of U.S.	*“I would say that [for some] cancers*, *we haven't [ordered NGS panels] as much because…[we may only need] individual mutation testing*.*”*
Quote P: Interviewee 4, male, in practice for more than 20 years, estimates sending between 10 and 20 samples for testing monthly from a VA site in west region of U.S.	*“[We frequently deal with the question of] should we do a targeted panel for EGFR or ALK [for lung cancer]*, *or*, *should we send it out for a more comprehensive [assessment through the NPOP]*.*”*
Quote Q: Interviewee 2; male, in practice between 10 and 20 years, estimates sending ~8 samples for testing monthly from a VA site in midwest region of U.S.	*“[As a thoracic oncologist*, *we want the results] to [show EGFR*, *ALK*, *ROS-1 and]…B-RAF…[I’d like to see] some of the immunotherapy data*, *that it's nice to see micro-satellite assessment*. *I'd like to see more*.*”*
Quote R: Interviewee 3; male, in practice between 10 and 20 years, estimates sending between 5 and 10 samples for testing monthly from a VA site in west region of U.S.	*“As Section Chief [of Hematology-Oncology*, *I met with]…the head of pathology [and we] sort of decided that if people are ordering piecemeal tests that are included in [NGS panels]…we're going to [ask them to] change [their orders to simply NGS]*.*”*
Theme 4: The Availability of the NPOP Has Expanded Options for Select Patients	
Quote S: Interviewee 20; male, in practice between 10 and 20 years, estimates sending between 5 and 10 samples for testing monthly from a VA site in west region of U.S.	*“[Now if we find a mutation*, *we will] be treating a patient with a very specific drug for that particular mutation*. *So it definitely makes a big difference*, *because now a lot of mutations are found*. *There are [currently] more and more mutations we come across*.*”*
Quote T: Interviewee 11; female, in practice for less than 10 years, estimates sending ~2 samples for testing monthly from a VA site in midwest region of U.S.	*“I definitely have had a couple [patients for which early sequencing] changed our management…Had we waited and considered [therapy without sequencing first]*, *like [chemotherapy]*, *we [would] have gotten…[less] benefit of [targeted therapy in the front-line setting]*.*”*
Quote U: Interviewee 19; female, in practice between 10 and 20 years, estimates sending between 5 and 10 samples for testing monthly from a VA site in south region of U.S.	*“So I had a patient with metastatic esophageal cancer who had failed all initial lines of therapy*. *We sent them for next-gen sequencing*, *and he had MSI-high [disease]…so I gave him immunotherapy*.*”*
Quote V: Interviewee 1; female, in practice for less than 5 years, estimates sending ~8 samples for testing monthly from a VA site in west region of U.S.	*“[I sent testing for four patients]*. *I got answers for all of them*, *and it was helpful for at least*, *probably*, *three of them*.*”*
Quote W: Interviewee 13; female, in practice between 10 and 20 years, estimates sending between 2 and 5 samples for testing monthly from a VA site in east region of U.S.	*“I [feel that most results] come back with [driver mutations] that are not [currently actionable]*, *but I do expect that to change in the future as more data and [mutational profiles] are accumulated*.*”*
Quote X: Interviewee 20; male, in practice between 10 and 20 years, estimates sending between 5 and 10 samples for testing monthly from a VA site in west region of U.S.	*“I will [say] that probably most of [the reports] come back with…targets that are not usable but I do expect that to change in the future as more data and…[knowledge of]… more [mutations]…are accumulated*.*”*
Theme 5: The Completion of Tumor DNA Sequencing through the NPOP Could Help to Improve Research Efforts within VHA Oncology Practices	
Quote Y: Interviewee 10; female, in practice for less than 10 years, estimates sending ~2 samples for testing monthly from a VA site in midwest region of U.S.	*“I'd love for [the automated reports or NPOP consultation service] to be able to point out*, *‘Here's a trial at this VA center that this veteran might be eligible for’ [based upon the identified mutations]*.*”*
Quote Z: Interviewee 7; female, in practice between 1 and 9 years, estimates sending 1 sample or less for testing monthly from a VA site in south region of U.S.	*“I think from an academic perspective*, *it's always very interesting to see what mutations are identified…Sometimes [these mutations] also correspond with ongoing trials*.*”*
Quote AA: Interviewee 6; female, in practice between 10 and 20 years, estimates sending ~1 sample for testing monthly from a VA site in east region of U.S.	*“That's a big thing [with comprehensive NGS panels]*,*is the clinical trial part of it*. *That's a missing link…[we need to have trials readily available at the VA based upon the results of testing]”*
Quote AB: Interviewee 18; male, in practice for more than 20 years, estimates sending 1 sample or less for testing monthly from a VA site in east region of U.S.	*“So I think [that our comprehensive] data [involving veteran patients]… might be more valuable than clinical trials*, *you know*? *There is nothing [better] between a clinical trial-based outcome from a group of patients and this situation*, *where you [deliver targeted therapies in a real-world setting]*.*”*
Quote AC: Interviewee 3; male, in practice between 10 and 20 years, estimates sending between 5 and 10 samples for testing monthly from a VA site in west region of U.S.	*“[One of my expectations] would be more efficient [use of] the available tissue*, *and that hopefully*, *[widespread sequencing] would provide some nice database for the VA nationally for research purposes*.*”*

EGFR: Epidermal Growth Factor Receptor; ALK: Anaplastic Lymphoma Kinase; MSI: Microsatellite Instability

### Theme 1: Educational efforts regarding tumor DNA sequencing should be undertaken

Nearly all interviewees discussed their desire to develop a greater understanding of at least one of three key facets of tumor DNA sequencing. First, oncologists raised concerns that they were unsure of the optimal forms of tumor samples and the necessary steps to prepare samples to conduct NGS (Table 2, Quotes A and B). Interviewees frequently expressed that certain limited forms of tumor sampling, such as core needle biopsy or fine needle aspiration, may yield insufficient tissue for analysis. Since these low-volume samples are also being used for other forms of precision testing, namely IHC staining for Programmed-Death Ligand 1 (PD-L1) status, the amount of available tissue may be further reduced prior to submitting for NGS. Second, oncologists detailed their interest to learn more about the identified mutations, specifically rare or atypical ones, within genes that are associated with therapies that are currently approved by the US Federal Food and Drug Administration (FDA) (Table 2, Quotes C–E). VHA Oncologists are provided with interpretative summaries from the external vendor laboratory, which discusses the current literature regarding the discovered mutations, and they may also reach the NPOP national consult service with further questions. Nonetheless, the interviewees were concerned about their overall lack of familiarity with the depth of information being provided and would prefer to have a stronger foundational understanding of uncommon gene variants. Last, a few interviewees shared an interest in a more expansive knowledge of the nuances related to the biochemical principles behind NGS (Table 2, Quote F). The science and terminology of NGS is relatively new to many clinicians, and some form of educational initiatives led by genomic experts may help oncologists develop a more thorough understanding of this state-of-the-art diagnostic assessment.

### Theme 2: Pathology departments share a critical role in facilitating test completion

Fourteen (70%) interviewees accentuated the value of their relationships with pathologists who are affiliated with their local VHA sites to facilitate and expedite the completion of testing through the NPOP. Interviewees felt that requests for multi-gene NGS assessments were efficiently conducted when their associated pathologists believed in the utility of tumor DNA sequencing for those respective cases (Table 2, Quotes G and H). Under these circumstances, oncologists could simply message their respective Pathology Departments by electronic mail or through the electronic medical record, and pathologists would then oversee the completion of each necessary step to prepare and submit tumor samples. Contrarily, interviewees felt reticent to order testing when their associated pathologists did not confidently believe in the prognostic and/or therapeutic roles of comprehensive multi-gene NGS panels. A majority of interviewed oncologists commented that meeting with the leadership of their respective Pathology Departments to detail a streamlined approach to complete testing was highly beneficial (Table 2, Quotes I and J). These interviewees felt that their requests for testing were less likely to be postponed once a systematic process was well-defined in conjunction with their affiliated pathologists. Finally, a substantial minority of oncologists were concerned that the rising number of orders for tumor DNA sequencing has been burdensome for the technicians responsible for preparing and submitting samples (Table 2, Quotes K and L). The size of staff within Pathology Departments is often stable yet the orders for tumor DNA sequencing have been growing, thereby leading to additional work for a finite number of personnel.

### Theme 3: Tumor DNA sequencing via NGS Serves as the most comprehensive testing modality within precision oncology

Interviewed medical oncologists frequently commented on the expansiveness of the results provided by multi-gene NGS assessments. The breadth of evaluated genes is unique compared to other sequencing modalities (Table 2, Quotes M and N). A few interviewees discussed the benefits of ongoing research—as the field continues to grow and our knowledge of certain mutations improves, the results from previous NGS assessments will be of greater value with a larger number of available targeted therapies. Interviewees also commonly discussed the role of NGS within the battery of tests available in precision oncology. Oncologists are now facing the issues of determining the optimal timing to order NGS and defining the appropriate sequence to conduct precision testing (Table 2, Quotes O and P). Since multi-gene NGS is now universally felt to be the most comprehensive test among interviewees, these oncologists are considering conducting NGS early in the course of a patient’s disease and using the modality as a singular replacement for all other forms of precision testing. A few interviewees raised the notion of standardizing precision testing practices at their local VHA sites to limit the completion of alternative tests that provide repetitive results (Table 2, Quotes Q and R). These oncologists detailed that some form of validation was required for multi-gene NGS panels to completely replace all other forms of precision testing. If this were to occur, oncology practices should globally seek to eliminate the completion of other forms of precision testing that duplicate the findings from multi-gene NGS assessments.

### Theme 4: The availability of the NPOP has expanded options for select patients

Eleven (55%) interviewees reported that conducting tumor DNA sequencing through the NPOP has led to changes in systemic treatment for patients managed at their respective VHA practices. A few interviewees shared experiences with prescribing targeted therapies in the frontline setting based upon the results of sequencing (Table 2, Quotes S and T). In these situations, NGS panels were completed shortly after the diagnoses of metastatic cancers were confirmed. As such, these oncologists were able to limit exposing their patients to traditional cytotoxic chemotherapy by choosing targeted approaches. Other interviewees shared their experiences with prescribing targeted agents for patients with malignancies that were refractory to initial systemic treatments (Table 2, Quotes U and V). Under these circumstances, oncologists felt that targeted therapies provided hope to patients who were not responding to initial systemic chemotherapy. Lastly, even among interviewees who had not yet prescribed treatments based upon the results of tumor DNA sequencing, the availability of the NPOP provides them with optimism that the outcomes among their patients will improve in the future through sequencing (Table 2, Quotes W and X).

### Theme 5: The Completion of tumor DNA sequencing through the NPOP could help improve research efforts within VHA oncology practices

A substantial minority of interviewees commented on the potential impact of the NPOP towards expanding research efforts both within individual VHA oncology practices and throughout the VHA. Interviewees frequently conveyed excitement that the identification of a large number of actionable mutations among veterans with cancer would lead to a rapid growth in enrollment within clinical trials at individual VHA sites (Table 2, Quotes Y—AA). Among these interviewees, a small number were concerned that clinical trials may not be accessible to some veterans who were found to have actionable mutations but were receiving care at VHA sites in remote areas. Next, 6 (30%) oncologists discussed the possibility to conduct comprehensive investigational evaluations based upon the large mutational database that could be developed through the NPOP (Table 2, Quotes AB and AC). Such a database could be used to examine common mutational pathways among veteran patients with cancer and to review responses to targeted therapies in a real-world setting. The results from these possible studies would further help to improve the collective current understanding of precision oncology while providing specific nuances on the behalf of veterans with metastatic malignancies.

## Discussion

The summative collective findings from our interviews indicate that VHA oncologists seek a greater foundational understanding of tumor DNA sequencing, pathologists serve an important role towards facilitating the completion of testing, multi-gene NGS panels are felt to be the most comprehensive diagnostic assessments within precision oncology, and the NPOP provides VHA oncologists with confidence that the outcomes of their veteran patients will improve by not only offering access to targeted therapies but also encouraging further research initiatives. Our semi-structured interviews were conducted among a diverse set of VHA oncologists who served within different areas of the US and represented varying levels of experience. The interviewees expounded on a wide breadth of issues surrounding tumor DNA sequencing, ranging from the process to logistically request NGS for an individual patient to the potential investigational benefits of sequencing thousands of patients across the VHA.

### Previous assessments of oncologists’ perceptions of tumor DNA sequencing

Within the VHA, Arney et al. previously interviewed 30 oncologists to understand the perceived barriers to completing tumor DNA sequencing for patients with metastatic non-small cell lung cancer (NSCLC) [17, 18]. The authors observed a wide spectrum of practices and confidence in conducting sequencing. Their findings are in contrast to the results of our semi-structured interviews, which frequently conveyed optimistic feelings towards the roles of the NPOP and multi-gene NGS assessments. A few caveats must be raised while comparing their findings to our results. First, sequencing for specific mutations among patients with metastatic NSCLC generally involves single-gene or small multiplex PCR panels, which outputs far less genomic information than multi-gene NGS panels [19]. As such, the volume of results is exponentially less, and if the likelihood of identifying an actionable mutation is minimal, which is often the case among veterans who are former or current smokers, the utility of sequencing one or a few genes for patients with NSCLC may not be as fruitful as completing comprehensive NGS assessments for patients with other metastatic solid tumors [20]. Next, the authors completed their interviews by early 2016, and as precision oncology further integrates into clinical practice, medical oncologists have likely developed a greater familiarity with tumor DNA sequencing in the past few years, thereby leading to a more favorable impression of conducting testing.

External to the VHA, two investigations have quantitatively assessed oncologists’ perceptions of tumor DNA sequencing via NGS for patients with metastatic solid tumors. First, Gray et al. surveyed 160 physicians at an academic institution between 2011 and 2012 prior to the center making a 41 gene panel routinely available to clinicians [21]. Interestingly, the participants conveyed a wide range of confidence in their understanding of testing results, with a substantial percentage of cancer specialists feeling uncomfortable with their current level of knowledge of molecular sequencing. Though our interviews were conducted approximately six years after the work by Gray et al., we found that medical oncologists working within VHA practices are still concerned that more education is warranted with the increasing use of testing. Second, Johnson et al. recently surveyed 52 pediatric oncologists at an academic center prior to instituting an enterprise-wide NGS panel [22]. Only 35% of pediatric oncologists reported feeling comfortable in understanding and discussing the results of sequencing for somatic mutations with patients while 27% felt comfortable with the results of testing for germline mutations. Despite the clear data illustrating the lack of familiarity with the roles and results of tumor DNA sequencing via NGS among oncologists, these institutions, like other public and private cancer centers across the country, have widely implemented and accelerated their sequencing programs.

### Implications of findings

As conveyed by Theme 1, the participants in our study shared a wide range of concerns related to their understanding of the steps required to conduct and implement tumor DNA sequencing, ranging from the procedural requirements to efficiently conduct NGS to interpreting the reported results on behalf of their patients. As of now, centers are integrating advisory support panels into their precision oncology programs with the hopes of addressing these widespread queries among oncologists [23–25]. Similarly, the NPOP has developed a monthly molecular tumor board in which oncologists serving within VHA practices throughout the country may discuss atypical mutations and specific cases with experts in molecular biology. Though the program’s monthly tumor board has counseled oncologists with specific cases, it certainly does not satisfy the desire of a few interviewees in our study who requested for a far greater foundational knowledge of tumor DNA sequencing, which most likely requires some form of lecture-based curriculum delivered by genomic experts. Consequently, wider educational efforts designed to develop and refine oncologists’ knowledge of sequencing should be considered.

Our second theme suggests that the traditional distinction between the roles of clinicians and those of pathologists may need to be reconsidered in this modern era of precision oncology. Generally, diagnostic testing is managed by pathologists while systemic treatments are administered by medical oncologists. The separation between the two disciplines may now be blending with the rapid integration of molecular testing into routine clinical practice. Medical oncologists are desiring a greater knowledge regarding the modalities of precision testing, and pathologists are seeking an enhanced understanding of the clinical relevance for the recent rise in requests for tumor DNA sequencing [26]. Strengthening the collaboration through shared knowledge between oncologists and pathologists will not only improve the fund of knowledge among both sets of physicians but also complement the approach to each patient with metastatic cancer. Efforts to further integrate pathologists into the delivery of patient care have been undertaken, and future work should seek to augment oncologists’ communication with their affiliated Pathology Departments [27].

Theme 3 conveys a common interest among our interviewees for a more streamlined approach to precision testing. As of now, the number of assessments related to precision oncology can be significantly complex for clinicians [28]. NGS of large multi-gene panels provides hope to oncologists that the number of alternative tests required for patients with metastatic solid tumors may be reduced in the future. Unfortunately, the current interval of time required to complete comprehensive NGS panels, the amount of tissue necessary to conduct testing, and the inability of sequencing to identify the results of certain tumor biomarkers, namely PD-L1 status and uncommon translocations, has restricted its use as the singular diagnostic test encompassing all clinically relevant alterations within the field of precision oncology. Nonetheless, further investigations may improve the utility of NGS to predict responses to a large majority of systemic therapies. Specifically, the identification of more prognostic and predictive mutations may allow for NGS to serve as an appropriate replacement for a collection of alternative forms of precision testing.

The fourth and fifth themes that were observed in our study illustrate the interviewees’ level of confidence in the summative utility of tumor DNA sequencing. Previous work has shown that the rate of actionable somatic mutations associated with FDA-approved therapies among patients with solid tumors is relatively low depending on the cancers assessed [3]. Despite the evidence reporting a limited benefit for routinely conducting sequencing via comprehensive multi-gene NGS panels, our interviewees commonly felt that sequencing is associated with improved outcomes for their patients. In addition, our interviewees were optimistic that the rising number of samples undergoing sequencing will ultimately lead to the development of more targeted treatments and encourage the expansion of available clinical trials across the VHA. Our findings therefore indicate that oncologists believe in the utility of tumor DNA sequencing among patients with various metastatic solid tumors, which further substantiates the role for NGS in clinical practice. Consequently, cancer practices throughout the country should continue preparing for the widespread growth of molecular sequencing.

In accordance with the expansion of NGS practices, further clinical studies will be required to validate the widespread use of tumor DNA sequencing among patients with metastatic solid tumors. Such studies could assess survival outcomes, changes in treatment practices, and/or enrollment in ongoing clinical trials. These studies are needed to validate our interviewees’ perceptions that outcomes are improved with tumor DNA sequencing and the number of completed clinical trials has grown with incorporating multi-gene NGS panels into practice.

### Limitations

A few key limitations of our qualitative study must be discussed. First, our interviewees solely included medical oncologists who served veterans within the VHA. Though many of the perceptions and concerns surrounding tumor DNA sequencing are likely similar between oncologists within the VHA and those employed elsewhere, the familiarity of sequencing may vary across practice settings, and the availability of consultative support similar to that provided by the NPOP is potentially unavailable to oncologists external to the VHA. Therefore, the implications of our findings may not be universally applicable. Second, our purposive sampling approach only sought participants who had requested at least one sample for sequencing through the NPOP. Oncologists who have never requested testing may have a lesser understanding of the indications and benefits of tumor DNA sequencing, and their input could have been valuable to further highlight the need for specific educational initiatives. In addition, oncologists who had not previously ordered testing may have felt that completing multi-gene NGS panels was simply not beneficial, and their perceptions regarding the utility of tumor DNA sequencing could have been critically missed. Third, though our interviewees provided important information about the vital role of pathologists towards the completion of molecular sequencing, we excluded providers who were not medical oncologists from our study, and consequently, the perceptions of pathologists were not included. A separate qualitative analysis is warranted to assess the opinions and concerns regarding the rising number of requests for multi-gene NGS panels among this critical group of physicians.

## Conclusions

In this qualitative study, we found that medical oncologists serving within the VHA commonly believe that tumor DNA sequencing through the NPOP leads to an improvement in outcomes for patients suffering from metastatic solid tumors. Though the oncologists participating in this study expressed positive feelings regarding the utility of tumor DNA sequencing, no clinical data has confirmed improvements in survival with widespread NGS practices. Furthermore, oncologists desire more education from technicians and genomic experts to better understand the requirements for sample preparation and to comfortably interpret the findings from NGS reports. The process to efficiently conduct testing for a large volume of patients is optimized through effective working relationships between medical oncologists and pathologists. Ideally, all of the clinically relevant biomarkers within the field of precision oncology may be identified by ordering a comprehensive large NGS panel, but currently, NGS is an additional diagnostic assessment within the battery of precision tests. As the use of tumor DNA sequencing continues to grow, these findings should be taken into consideration as oncologists are chiefly responsible for ordering NGS panels and prescribing treatments based upon the results.
